# Allergic asthma manifestations in human and seropositivity to *Toxocara*, a soil-transmitted helminth of carnivores: A case-control study and scoping review of the literature

**DOI:** 10.3389/fmed.2022.920182

**Published:** 2022-09-29

**Authors:** Nasrin Bazargan, Azadeh Nasri Lari, Mehdi Borhani, Majid Fasihi Harandi

**Affiliations:** ^1^Department of Pediatrics, Afzalipour Medical Center, School of Medicine, Kerman University of Medical Sciences, Kerman, Iran; ^2^School of Medicine, Fasa University of Medical Sciences, Fasa, Iran; ^3^State Key Laboratory for Zoonotic Diseases, Key Laboratory of Zoonosis Research, Ministry of Education, Institute of Zoonosis, College of Veterinary Medicine, Jilin University, Changchun, China; ^4^Research Center for Hydatid Disease in Iran, School of Medicine Kerman University of Medical Sciences, Kerman, Iran

**Keywords:** pediatric asthma, toxocariasis, scoping review, publication trend, soil-transmitted helminths, adulthood asthma

## Abstract

Asthma is a common respiratory disease affecting humans. Helminth parasites, including *Toxocara* species, have been implicated as predisposing factors of asthma. However, various studies present different findings on asthma-*Toxocara* association. Herein, we investigated the association of asthma manifestations with *Toxocara* seropositivity in a case-control setting on 248 participants (147 women and 101 men), with 124 healthy individuals as the control group and 124 patients known to have asthma based on the medical records of asthma clinics of Kerman University of Medical Sciences. Consequently, we presented a scoping review of all previous studies carried out on this topic, summarizing current findings and existing knowledge on this issue. Of 248 participants, 31 (12.5%) were *Toxocara*-seropositive, of which 19 (15.3%) were in the patient group and 12 (9.7%) in the control group. A significant relationship was found between asthma severity and age in *Toxocara*-seropositive individuals (*P* < 0.04). We found no significant relationship between asthma and *Toxocara* seropositivity. We identified 7,724 related records in three major scientific databases, NCBI PubMed, Scopus, and Google Scholar. The review of the literature showed that there are 80 published articles on asthma-*Toxocara* relationship with contradictory findings. More than half of the studies were performed in only four countries, namely, Brazil, the Netherlands, the United States, and Iran. The study population in 70% of the studies were children, and few studies investigated asthma-*Toxocara* association in adults. The most common study designs for investigating the association of asthma and *Toxocara* seropositivity were cross-sectional (35.0%), case-control (27.5%), and animal experimental (12.5%) studies. This study found no significant relationship between asthma manifestations and toxocariasis in a case-control setting. However, a scoping review of the current literature suggests that further experimental and field longitudinal cohort studies are required to elucidate the nature of asthma-*Toxocara* interaction in humans.

## Introduction

Asthma, a common inflammatory disease of the respiratory tract, is considered a major public health problem in the adult population as well as a chronic and sometimes life-threatening illness in children, causing negative effects on the socioeconomic status and the quality of life of patients (1). In the past four decades, the prevalence of asthma has increased in all countries. The World Health Organization (WHO) estimated that about 300 million people suffer from the disease worldwide. In addition, the global annual asthma burden is estimated at 250,000 deaths and 15 million disability-adjusted life years (DALYs) (2). Asthma is the most common chronic illness in the pediatric population. In two separate studies, the prevalence of asthma in Iranian adults and children was estimated at 8.9 and 4.4%, respectively (3, 4).

The main etiology of asthma is not completely understood, but there is evidence indicating that asthma is caused by a combination of genetic and environmental factors. Indoor and outdoor allergens, air pollution, cold air, chemical irritants, extreme emotional arousal, and pathogens are among the environmental factors of asthma. However, family history is one major genetic determinant of the disease, with several genes, such as ADAM33, GSTM1, and LTC4S, being implicated (5, 6). Therefore, investigating the nature and significance of risk factors involved in asthma development in adults and children is essential (7).

There is evidence indicating that *Toxocara, Anisakis*, and *Ascaris* species are risk factors for asthma in human populations (8). Meta-analysis studies revealed a relationship between the presence of Ascarid geohelminths and asthma (9). Helminthic infection (HI) is capable of stimulating host inflammatory responses against the parasite. Inflammation plays a major role in the pathophysiology of asthma. Inflammatory cells (eosinophils, macrophages, dendritic cells, lymphocytes, etc.) and inflammatory mediators including IL4, IL5, IL9, IL13, IL15, IL16, and IL17A-&F, as well as IgE, are immunologic factors involving in this disease (10). HI in humans is associated with the presence of CD4 T cells, IgE increase, and subsequent production of cytokines, including IL5, IL-4, and IL-13. Some epidemiological and experimental studies suggested that HI, particularly *Toxocara* spp., promotes the development of asthma (11).

Toxocariasis is one of the neglected tropical diseases with undetermined public health and economic impacts (12). *Toxocara canis* and *Toxocara cati* are intestinal nematodes of dogs and cats, which cause human toxocariasis, a cosmopolitan helminthic zoonosis. Human is considered an accidental host that acquires infection by ingestion or inhalation of infective eggs of *Toxocara* spp. in soil or other contaminated materials (13). Migration of *Toxocara* larvae into various human body organs can cause many problems, including visceral larva migrans (VLM), ocular larva migrans (OLM) syndrome, and covert toxocariasis (CT) syndrome (14). Associated symptoms of CT in children include cough, pharyngitis, wheezing, pneumonia, and asthma-like symptoms.

According to many studies in the world, the frequency of soil contamination with *Toxocara* eggs varies in different areas, ranging from 6.6 to 87.1% (15). The rate in Iran has been reported as 21.6% (16, 17). Seroepidemiological studies in different countries indicate the worldwide distribution of toxocariasis. The results of a systematic review in Iran showed a seroprevalence of 15.8% in humans (18).

Many studies examined the association of *Toxocara* infection with asthma and/or bronchial hyperresponsiveness (BHR) (19, 20). However, conflicting results have been reported from different parts of the world. Several studies indicated a positive correlation between asthma and *Toxocara* serology, while no significant association has been reported in several other studies (8, 9, 16). The complex interactions of the immune system and hypersensitivity reactions, the hygiene hypothesis, and differences in the genetic makeup and socioeconomic variables can be considered the significant causes of conflicts in the results of studies correlating helminthic infections with autoimmune diseases and asthma (21–25).

In the literature review, the authors noted that most of the studies focused on childhood asthma, and fewer studies have been conducted on the association between asthma and toxocariasis in adult populations (26). Therefore, to elucidate the significance of toxocariasis in the development of asthma and airway allergy, more human and animal studies are required to be conducted on this issue in different geographical areas of the world. In the present study, we investigated the association of allergic asthma manifestations with positive *Toxocara* serology in southeastern Iran. In addition, we performed a scoping review on previous studies carried out on the association between asthma and toxocariasis, presenting current literature findings and global distribution of knowledge on this issue.

## Materials and methods

### Case-control study

The study protocol was reviewed and approved by the Research Ethics Committees of Kerman University of Medical Sciences (REC code 99000235). The participants in the survey were informed of the main research goals and signed a written consent form before sampling. This research was performed on 248 participants (147 women and 101 men), with 124 healthy individuals as the control group and 124 patients known to have asthma based on the medical records of asthma clinics of Kerman University of Medical Sciences, as well as one private allergy and asthma clinic in the city of Kerman. The participants were clinically evaluated for asthma signs and symptoms by the sub-specialist author (NB) according to the current guidelines on asthma management and prevention (27). The exclusion criteria were allergic conditions other than respiratory allergies and people with occupational lung diseases causing asthma-like pulmonary symptoms. The control group consisted of 124 individuals with no history of asthma and/or allergies, referring to clinical laboratories for routine annual checkups. Participants' data were collected by two investigators through physical examination of the patients and by conducting history and careful interviews with the parents or guardians of the patients. Data were collected by standard pre-coded questionnaires for each participant, including name, age, sex, place of residence, education, and duration of asthma and allergic manifestations (for the patient group). The average age of the participants in the case and control groups was 31.01 and 29.65, respectively (*P* > 0.05).

According to asthma guidelines, individuals with allergic manifestations were divided into four groups based on the severity of asthma by an asthma sub-specialist (27). The patients were clinically divided into four groups, namely, intermittent, mild persistent, moderate persistent, and severe persistent according to the frequency of symptoms, forced expiratory volume in 1 second (FEV1), and the peak expiratory flow rate (28).

A 5 mL blood sample was collected in a tube without anticoagulant from each participant in the scheme. The serum was separated and stored at −70°C until use. Patient and control samples were serologically evaluated by using a *Toxocara canis* IgG-ELISA kit (IBL, Hamburg, Germany) with a sensitivity and a specificity of 96.9 and 98.6%, respectively. ELISA was performed according to the manufacturer's instructions. Information from the questionnaire and the result of the serological test were finally entered into MS Excel software.

### Statistical analysis

Data analyses were conducted by SPSS version 22 statistical software. Descriptive statistics were applied to express the data as frequencies and percentages. The main dependent and independent variables include the presence of asthma and *Toxocara* serology, respectively. For categorical variables, the difference between the groups was tested by using the chi-square test. For continuous variables, Student's *t*-test or one-way ANOVA was used to examine whether the differences in the mean values of variables between the case and control groups were statistically significant. The *p*-values < 0.05 were considered statistically significant. Also, the relationship between the prevalence of severity of asthma/allergy and age groups was examined for the studied groups. Further analysis was carried out to map findings of the current literature and the global distribution of knowledge on this topic using MS Excel and ArcGIS software.

### Scoping review of the literature

We performed a systematic search following the guidelines provided by the Preferred Reporting Items for Systematic Reviews and Meta-Analyses (PRISMA) (29–32). In the current study, principal data sources were obtained from the literature. We searched three major science databases, namely, NCBI PubMed, Scopus, and Google Scholar. Using different combinations of the search terms [“asthma” AND (“toxocara” OR “toxocariasis”)] in title, abstract, or keywords, all the articles published until 8 May 2020 were retrieved and stored in an MS Excel file. Custom search strings were used for each database to reduce the number of irrelevant hits and increase search accuracy. The following inclusion criteria were considered: articles with an English title and articles investigating asthmatic conditions, and *Toxocara* infection. The articles studying respiratory disorders other than asthma and articles studying other soil-transmitted helminths including *Ascaris, Trichuris*, and *Strongyloides*, or hookworm infections were excluded from the study. Also, opinion communications, editorials, perspectives, and letters were excluded. English articles were included in the study. Among non-English articles, only articles with English abstracts were included in the study. If needed, the full texts of non-English articles were translated using Google Translate.

After clearing the original data file, 6,331 of 7,724 studies were selected from. Only articles with the main topic related to the association of asthma and *Toxocara* infection were included in the study. Titles and abstracts of the studies were checked for relevance to the topic by two persons. Those studies considered relevant by both the researchers were automatically moved to the secondary sorting. A third researcher organized the studies and examined those articles considered relevant by only one of the two researchers. The studies identified as relevant by the third researcher were added to the secondary screening. Following the secondary screening, the full text of the selected documents was examined further to select eligible studies. All relevant studies of the secondary screening process were combined into a single dataset (178 studies) for use in the next stage.

During the next screening stage, the two researchers independently sorted out all the studies related to the initial sorting process using abstracts (if there were no abstracts, sections of results and conclusions were used to determine relevance). In the final sorting process, two researchers independently read the full text of all studies. The studies deemed relevant by the two researchers proceeded to the data extraction process, while studies deemed irrelevant were excluded. Studies with conflicting evaluations were discussed by the three researchers until an agreement was reached. Studies selected from this multi-stage systematic sorting process were included in the data extraction process.

In the final stage, the Excel spreadsheet was filled with data extracted from the eligible studies. For each article, different characteristics, including author name, journal title, year of publication, country, article type, number of citations, age-group, male/female, research methodology (cross-sectional, case-control, cohort, animal experiments, systematic review, and meta-analysis), statistical significance, diagnostic techniques, sample size, and the mean age, were identified.

## Results

### The case-control study

Serological investigation revealed 31 (12.5%) participants out of 248 as *Toxocara*-seropositive, of which 19 (15.3%) cases were in the patient group and 12 (9.7%) in the control group. Statistical analysis indicates no significant association between allergic asthma manifestations and *Toxocara* seropositivity (*P* = 0.249). Also, there was no significant association between *Toxocara* serology with different age groups (Table 1). Patients with asthmatic conditions were more likely to be *Toxocara*-seropositive than the healthy controls (OR = 1.69, 95% CI = 0.78–3.64); however, this was not statistically significant.

**Table 1:** Age distribution of the individuals according to *Toxocara* serology.

**Age group**	**Toxocara seropositive**	**Toxocara seronegative**	**Total**	***P*** **-value**
5–15	9 (16.1)	47 (83.9)	56 (22.6%)	*P* = 0.792
16–25	1 (3.1)	31 (96.9)	32 (12.9%)	
26–35	8 (15.1)	45 (84.9)	53 (21.4%)	
36–45	6 (22.2)	21 (77.8)	27 (10.9%)	
46–55	3 (11.5)	23 (88.5)	26 (10.5%)	
56–65	2 (6.9)	27 (93.1)	29 (11.6%)	
>65	2 (8.0)	23 (92.0)	25 (10.1%)	
**Total**	31	217	248 (100%)	

A significant relationship was found between the asthma severity and age in *Toxocara*-seropositive individuals (*P* < 0.04). It was found that, with increasing age, the severity of asthma manifestations was higher in the patients with positive *Toxocara* serology (Table 2). Patients with asthma with seronegative findings demonstrated a significant association between education level and severity of allergic manifestations, as the level of education increases, the severity of allergic manifestations decreased (Table 3).

**Table 2:** Severity of asthma manifestations according to different age groups in individuals with positive *Toxocara* serology.

**Age group**	**Intermittent**	**Mild persistent**	**Moderate persistent**	**Severe persistent**	**Total**	***P*** **-value**
5–15	2 (10.53%)	1 (5.27%)	0	0	3 (15.8%)	*P* < 0.04
16–25	0	1 (5.27%)	0	0	1 (5.27%)	
26–35	0	1 (5.27%)	1 (5.27%)	0	2 (10.54%)	
36–45	2 (10.54%)	1 (5.27%)	2 (10.54%)	0	5 (26.33%)	
46–55	0	2 (10.53%)	2 (10.53%)	0	4 (21.06%)	
56–65	0	0	1 (5.27%)	1 (5.27%)	2 (10.54%)	
>65	0	0	2 (10.54%)	0	2 (10.54%)	

**Table 3:** Severity of asthma manifestations and education level according to the results of *Toxocara* serology.

	**Intermittent**	**Mild persistent**	**Moderate persistent**	**Severe persistent**	**Total**	***P*** **-value**
Toxocara negative						*P* < 0.02
≤Diploma	3 (2.85%)	23 (21.85%)	46 (43.7%)	5 (4.75%)	77 (73.15%)	
>Diploma	3 (2.85%)	13 (12.35%)	10 (9.5%)	2 (1.9%)	28 (26.6%)	
Total	6 (5.7%)	36 (34.2%)	56 (53.2%)	7 (6.65%)	105 (100%)	
Toxocara positive						*P* < 0.3
≤Diploma	4 (21%)	3 (15.8%)	6 (31.6%)	0	13 (68.4%)	
>Diploma	0	3 (15.8%)	2 (10.5%)	1 (5.2%)	6 (31.6%)	
Total	4 (21.06%)	6 (31.6%)	8 (42.1%)	1 (5.2%)	19 (100%)	

### Scoping review of the literature

**Figure 1:**
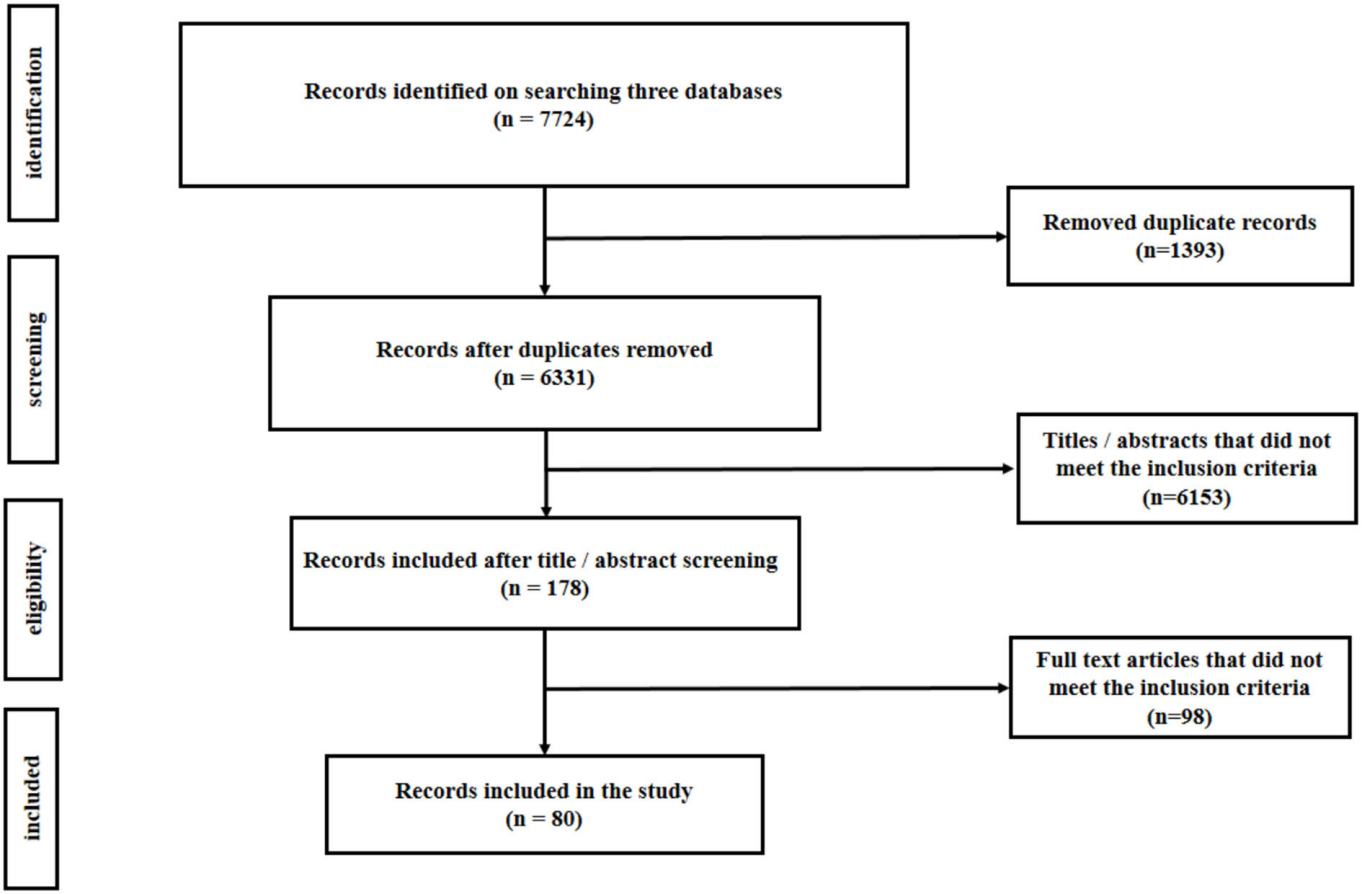
Flowchart showing the number of records retrieved at different stages of literature search.

Figure 1 shows the search results of the scoping review, describing the selection process of the records included in this study. In brief, 7,724 records were identified in the three major scientific databases, of which 1,393 studies were duplicate records. A total of 178 studies were selected after removing 6,153 articles not meeting the inclusion criteria according to the titles and/or abstracts. Finally, after reviewing the full texts of the articles, 98 studies did not meet the inclusion criteria, and 80 relevant records were included in the analysis.

**Figure 2:**
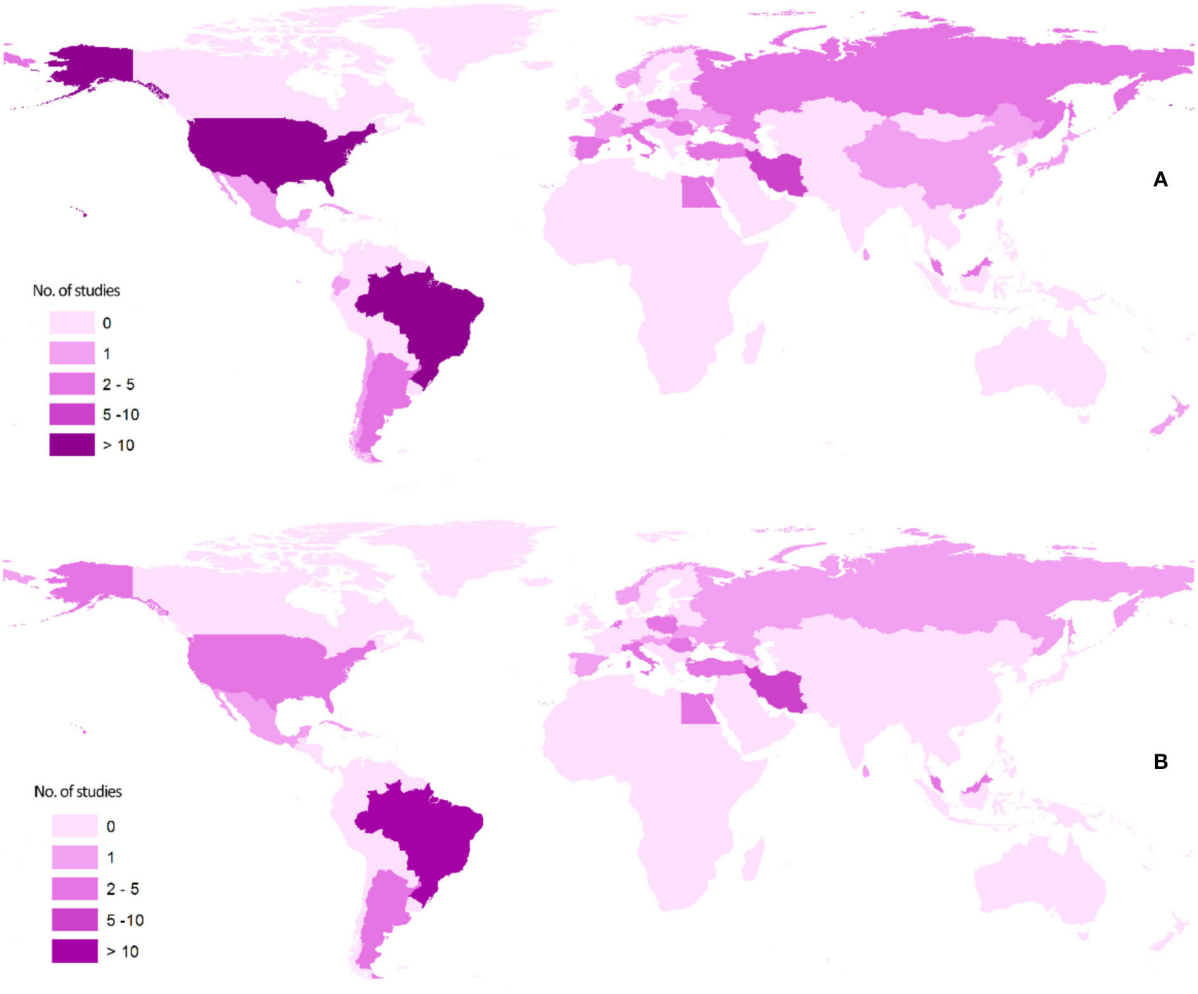
Maps showing the geographical distribution of the literature published on the association of asthma and *Toxocara* serology: **(A)** articles of all types and **(B)** analytical articles, that is, cross-sectional, case-control, and cohort.

Figure 2 shows the distribution of asthma-*Toxocara* articles published according to different geographical locations. Geographically, more than half of the studies were performed in only four countries, namely, Brazil, the Netherlands, the United States, and Iran.

**Figure 3:**
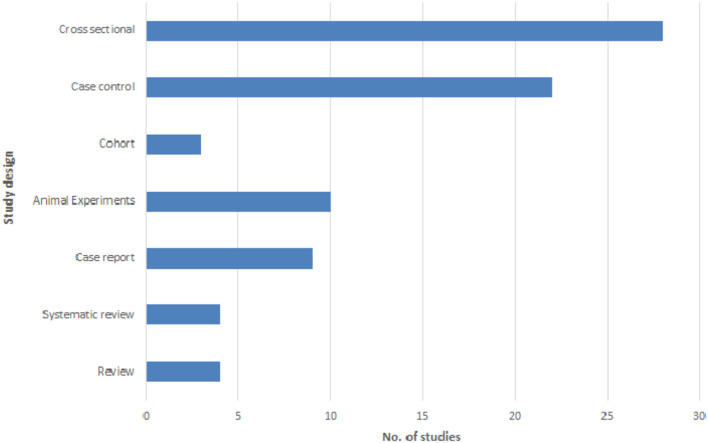
Frequency distribution of the studies investigating the asthma-*Toxocara* association according to different study designs.

The most common study designs for investigating the association of asthma and *Toxocara* seropositivity were cross-sectional (28/80, 35.0%), case-control (22/80, 27.5%), and animal experimental (10/80, 12.5%) studies. Other types of study designs included cohort studies, case reports, and reviews (Figure 3). It is noteworthy that the study population in 70% of the analytical articles (cross-sectional, case-control, and cohort) was children (Table 4).

**Table 4:** Summary of the analytical studies published on the asthma-*Toxocara* association according to different article features.

**Code**	**Country**	**Methodology**	**Sample size**	**Age group**	Significance^*^	**Citation**	**References**
1	United States	Case-control	176	Children	S	74	(33)
2	Brazil	Case-control	208	Children	NS	10	(34)
3	Malaysia	Case-control	124	Children	S	95	(35)
4	Romania	Case-control	164	Children	S	19	(36)
5	Egypt	Case-control	485	Both	S	21	(37)
6	Egypt	Case-control	680	Children	S	10	(38)
7	Sri Lanka	Case-control	196	Children	S	32	(39)
8	Brazil	Case-control	173	Children	NS	8	(40)
9	Malaysia	Case-control	45	Children	S	2	(41)
10	Iran	Case-control	100	Both	NS	2	(42)
11	Turkey	Case-control	53	Adult	S	30	(43)
12	Turkey	Case-control	184	Adult	NS	33	(44)
13	Egypt	Case-control	180	Children	S	5	(45)
14	Argentina	Case-control	100	Children	NS	13	(46)
15	Russia	Case-control	76	NA	NA	0	(47)
16	Argentina	Case-control	82	Adult	S	15	(48)
17	Iran	Case-control	86	Children	S	4	(49)
18	Mexico	Case-control	437	Children	NS	26	(50)
19	Egypt	Case-control	90	Children	S	45	(51)
20	Romania	Case-control	201	NA	S	0	(52)
21	Iran	Case-control	192	Children	NS	0	(53)
22	United states	Case-control	324	Children	NS	134	(54)
23	Iran	Cross-sectional	180	Children	NS	15	(55)
24	Poland	Cross-sectional	119	Children	ND	11	(56)
25	Brazil	Cross-sectional	237	Children	ND	8	(57)
26	Norway	Cross-sectional	435	Both	S-NS	12	(58)
27	Iran	Cross-sectional	630	Both	S	13	(59)
28	Romania	Cross-sectional	228	Children	ND	4	(60)
29	Netherlands	Cross-sectional	704	Children	S	10	(61)
30	Netherlands	Cross-sectional	1379	Children	S	203	(62)
31	Ukraine	Cross-sectional	50	Children	S	5	(63)
32	Brazil	Cross-sectional	606	Children	NS	43	(64)
33	Brazil	Cross-sectional	391	Children	NS	31	(65)
34	Spain	Cross-sectional	463	Adult	NS	67	(66)
35	Italy	Cross-sectional	753	Both	S	14	(67)
36	Netherlands	Cross-sectional	712	Children	S	142	(68)
37	Cuba	Cross-sectional	958	Children	S	29	(69)
38	Poland	Cross-sectional	26	Children	NS	1	(70)
39	Brazil	Cross-sectional	90	Children	S	67	(71)
40	Brazil	Cross-sectional	1148	Children	NS	71	(72)
41	United States	Cross-sectional	ND	Both	NS	2	(73)
42	Italy	Cross-sectional	336	Adult	ND	1	(74)
43	Iran	Cross-sectional	1150	Both	S	3	(75)
44	Iran	Cross-sectional	150	Children	NS	0	(76)
45	Brazil	Cross-sectional	791	Children	NS	23	(77)
46	Brazil	Cross-sectional	208	Children	S	6	(78)
47	Netherlands	Cross-sectional	234	Children	ND	3	(79)
48	United States	Cross-sectional	12174	Both	S	44	(80)
49	Austria	Cross-sectional	191	Adult	NS	25	(81)
50	Brazil	Cross-sectional	208	Children	S	78	(82)
51	Brazil	Cohort-study	1271	Children	NS	41	(83)
52	Hungary	Cohort-study	425	Children	S	25	(84)
53	Brazil	Cohort-study	77	Children	NS	7	(21)

* S = significant; NS, not significant; S-NS, significant in children-not significant in adults; ND, not determined.

**Figure 4:**
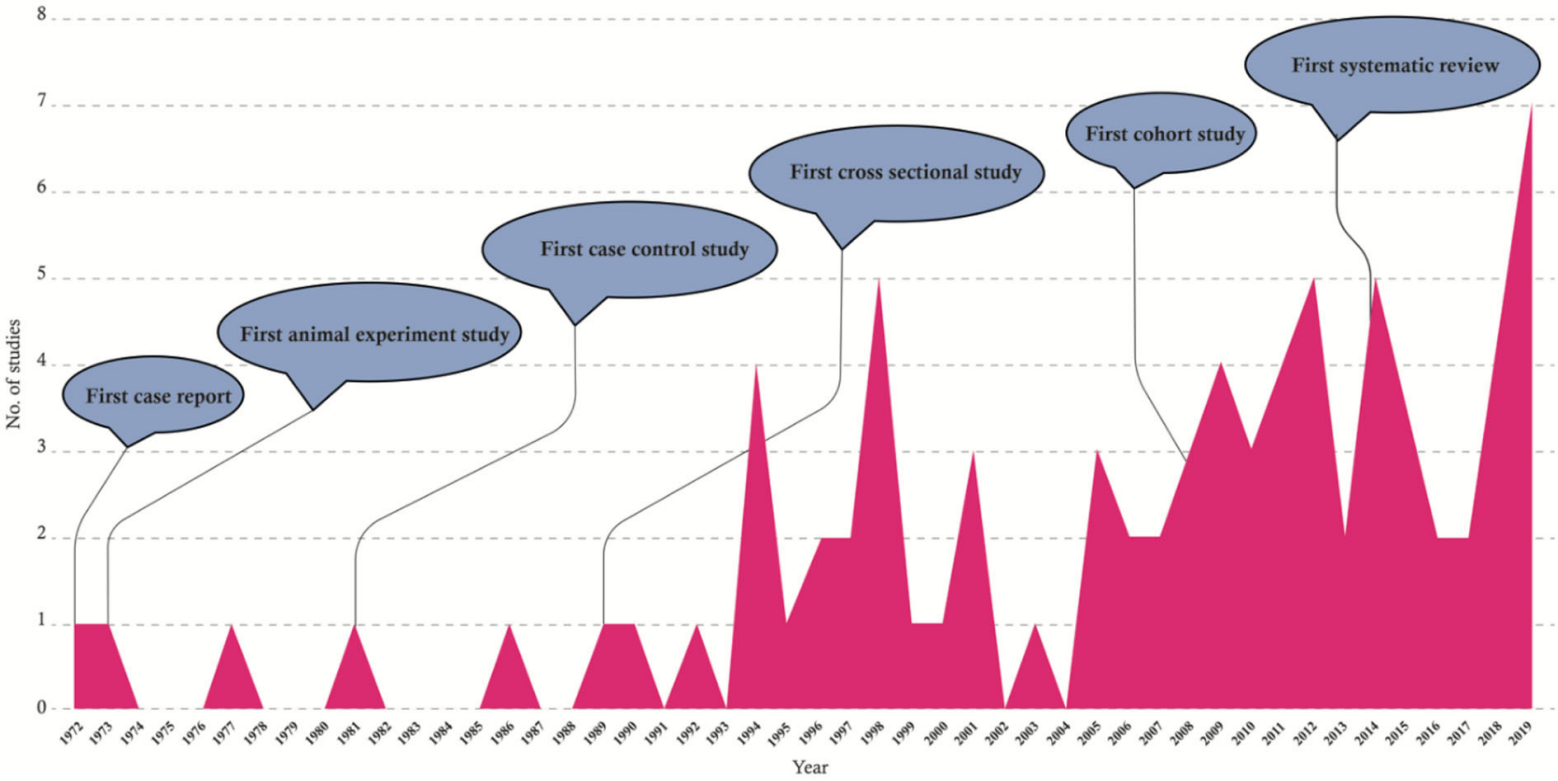
Graphical presentation of the time trends of the studies published on the asthma-*Toxocara* association from 1972 to 2019.

Figure 4 shows the time trends of asthma-*Toxocara* studies from 1972 to 2019. During the past three decades, these studies have been increasingly followed by different research workers around the world, and on average, 2.5 articles on this topic were published annually. The most frequently cited articles were reviews, systematic reviews, and cross-sectional studies, with 74.5, 45.5, and 33.3 citations per article, respectively.

## Discussion

The main purpose of our study was to investigate the probable association between asthma and *Toxocara* infection through a case-control study and a subsequent scoping review of the current literature examining the published evidence of the association between asthma and *Toxocara* seropositivity. Asthma is a chronic disease affecting children and adults worldwide, and a variety of environmental and genetic factors have been implicated in the development of the disease. House dust mite exposure, upper respiratory viral infections, and air pollution are among the probable environmental factors affecting asthma. Helminth infections are considered among the environmental factors associated with the development of asthma. In our study, although the likelihood of being *Toxocara*-seropositive in patients with asthmatic conditions was roughly more than that of the healthy controls, this presents no statistically significant association between *Toxocara* serology and asthma symptoms in this case-control setting. There are conflicting findings in the literature regarding the relationship between helminths and asthma. Several uncontrollable confounding factors can be implicated in this kind of study, including differences in diet and the genetic structure of the populations in various studies, other concurrent infections, and environmental pollutants. Also, different species of helminths demonstrated different associations with asthma and other allergic diseases. Some studies showed that anti-helminthic drugs for *Ascaris lumbricoides* are capable of reducing the symptoms of asthma (85, 86). There is evidence indicating that helminths can cause asthma and allergic manifestations in humans (8, 87, 88). Robust longitudinal cohorts are needed to address the correlation between helminth infections and the development of asthma and airway allergy and atopy.

The literature presents conflicting results regarding the association between asthma and toxocariasis. In our review of all case-control studies published to date, eight studies reported a non-significant and 13 studies reported a significant relationship between *Toxocara* infection and asthma; however, recent meta-analyses indicated a probable relationship between asthma and *Toxocara* serology (16, 26). Among three case-control studies conducted in different regions in Iran, two studies in the northern provinces of Alborz and Khorasan suggested no significant association between asthma and *Toxocara* serology; however, one study conducted in Isfahan, central Iran, indicated a significant association (42, 49, 53). Studies in Malaysia reported a significant relationship of allergic asthma manifestations with *Toxocara* seropositivity (35, 41). These studies were performed on children, while our study was conducted on all age-groups. This is particularly supported by the interesting study of Jogi et al. (58) in which the association between *Toxocara* serology and asthma was found significant in children, while no significant association was found in adults. It should be noted that 15 out of 22 published case-control studies were conducted on childhood asthma, and more studies are required for the adult population.

Several cross-sectional studies were conducted to describe the serum levels of anti-*Toxocara* antibodies in asthmatic individuals, showing different results in different geographical areas (Table 4). For instance, between 2016 and 2020, four studies were conducted on this topic in Iran, in which no significant asthma-Toxocara relationships were found in two studies, while the remaining studies indicate significant results (55, 59, 75, 76). The same is true in Brazil, where the data indicating significant and non-significant relationships were documented in three and four studies, respectively (57, 64, 65, 71, 72, 77, 78, 82).

Accidental ingestion of *Toxocara* spp. eggs leads to several syndromes in humans, and the ability of the helminth to cause VLM, OLM, neurological and cutaneous manifestations, and allergies is of interest to medical specialists. The spectrum of the disease manifestations from an asymptomatic infection to severe organ damage is related to the site of larval migration, parasite load, and inflammatory response in humans (13). The association of tissue helminth infections with peripheral blood eosinophilia, the presence of cytokines including IL-10 and IL-4, and elevated IgE levels are hypothetical pieces of evidence for the relationship between HI and asthma. *Toxocara* infection modulates the immune response to Th2 and the secretion of IL4, IL5, and IL13, which could theoretically be associated with asthma (89). However, protective effects of helminth infections in asthma symptom development have been documented in some studies. A nonsignificant increase in asthma risk has been reported by Leonardi-Bee et al. (9) in individuals infected with any helminth parasites. Nonetheless, they found that infection with *Ascaris lumbricoides* was significantly related to asthma, while hookworm infection was associated with significantly reduced risk of asthma. *Toxocara* infection in BALB/c mice models has shown respiratory morbidity and eosinophilic inflammation (90); however, more human and animal investigations are required to clarify mechanisms involved in airway hypersensitivity (83, 91).

According to a recent systematic review and meta-analysis, the global seroprevalence of *Toxocara* has been estimated to be 19.0% (92). In Iran, *Toxocara* seropositivity in humans has been reported as 1.7–33.7%, and the prevalence in dogs and cats has been estimated at 24.2 and 32.6%, respectively (93). The environmental contamination with eggs ranges from 1.7 to 63.3% in public parks and from 3.2 to 16.0% in vegetables (94).

In the present study, there were no significant relationships between age, sex, and literacy levels of the participants and *Toxocara* seropositivity, which was quite similar to the findings of the studies conducted in Malaysia, Nigeria, Turkey, and the United States (44, 54, 95). Nonetheless, Buijs et al. (62) indicated that *Toxocara* seropositivity was significantly lower in children younger than 10 years, which could explain the differences in the results (62). In previous studies, the association of age with the severity of allergic manifestations in asthma patients with positive *Toxocara* serology was not evaluated. In the present study, the severity of asthma manifestations increased with age among seropositive individuals (Table 2). Also, no significant relationship was found between the education level and severity of allergic manifestations in asthma among patients with positive *Toxocara* serology (Table 3). It is worth noting that a significant relationship between education level and the severity of allergic manifestations was observed among asthma patients with negative *Toxocara* serology.

Studies on the association between asthma and *Toxocara* seropositivity have been conducting for nearly half a century. The probable association of asthma with toxocariasis was investigated for the first time by Brown in 1972 who evaluated “Toxocaral antibody in nine asthmatic patients” (96). Subsequently, several animal experimental, case-control, and cross sectional studies were conducted until the new millennium. Nonetheless, most of the studies (65%) have been published in the last 15 years. In recent years, at least two studies have been conducted each year in this regard. Therefore, different types and levels of evidence are still lacking, and more focused animal investigations and extensive case-control and cohort studies are required to elucidate the role of *Toxocara* infection in the development of asthma in human.

More than half of the studies were performed in mainly four countries. As seen in Figure 2, most of the analytical studies are geographically restricted to America, Europe, and the Middle East. There is a large gap in knowledge on this topic in Africa and Asia, since very few studies were conducted in these regions, where many zoonotic helminths including *Toxocara* spp. are endemic.

Since 2010, conflicting results have been published regarding the association between asthma symptoms and *Toxocara* seropositivity. Interestingly, an equal number of articles proposed significant (14 records) and non-significant (14 records) associations between asthma and toxocariasis. Among 80 studies investigating the association of asthma with *Toxocara* seropositivity, 35 studies (43.8%) were significant, and 23 studies (28.7%) were not significant. In the remaining studies (mostly case reports, reviews, and cross-sectional studies), the significance values were not reported and/or not applicable (see the Supplementary file).

It is interesting to note that only a few studies investigated the association of asthma with *Toxocara* seropositivity in adults. The review of the literature revealed that 70% of the studies were exclusively conducted on children, and only 11.3 and 15.1% of the studies were performed in adults and all age groups, respectively. More studies are required on the probable role of toxocariasis in asthma development in the adult population. In addition, in-depth age-specific analyses should be performed on existing datasets to clarify the contribution of age in asthma-*Toxocara* interaction.

The most frequently cited articles were reviews, systematic reviews, and cross-sectional studies with 74.5, 45.5, and 33.3 citations per article, respectively. The three most cited articles were published in the Current Opinion in Allergy and Clinical Immunology (a review with 244 citations), European Respiratory Journal (a cross-sectional study with 203 citations), and American Journal of Epidemiology (a cross-sectional study with 142 citations). The average number of citations per article was 31.2. The total sample size of the cohort, case-control, and cross-sectional studies was 36,537, with an average sample size of 198, 591, and 1,086 subjects, respectively.

## Conclusion

Despite a multitude of studies conducted on the probable role of *Toxocara* infection on asthma development in different human communities, the level of evidence is not sufficient, particularly in adult populations, and further detailed animal investigations and extensive case-control and cohort studies as well as cellular–molecular explorations are required to elucidate the nature of asthma-*Toxocara* interaction in humans.

## Data availability statement

The original contributions presented in the study are included in the article/Supplementary material, further inquiries can be directed to the corresponding author.

## Ethics statement

The studies involving human participants were reviewed and approved by the Research Ethics Review Committee of Kerman University of Medical Sciences, Approval code: IR.KMU.REC.1399.285. Written informed consent to participate in this study was provided by the participants' legal guardian/next of kin.

## Author contributions

NB and MF: conceptualization and study design. AL and MB: data curation. NB, MB, and MF: data analysis and data validation. MF: funding acquisition. AL and MF: laboratory experiments. MB and MF: writing—original draft preparation. NB, AL, MB, and MF: revising and final approval of the manuscript. All authors contributed to the article and approved the submitted version.

## Funding

This study was supported by the Iranian Management and Planning Organization and Vice-Chancellor for Research, Kerman University of Medical Sciences (grant no. T.83/04).

## Conflict of interest

The authors declare that the research was conducted in the absence of any commercial or financial relationships that could be construed as a potential conflict of interest.

## Publisher's note

All claims expressed in this article are solely those of the authors and do not necessarily represent those of their affiliated organizations, or those of the publisher, the editors and the reviewers. Any product that may be evaluated in this article, or claim that may be made by its manufacturer, is not guaranteed or endorsed by the publisher.
