# Supplementary material for: Allergic asthma manifestations in human and seropositivity to Toxocara, a soil-transmitted helminth of carnivores: A case-control study and scoping review of the literature
Source: Front Med (Lausanne). 2022 Sep 29;9:920182. doi: 10.3389/fmed.2022.920182 (PMC9556890; doi:10.3389/fmed.2022.920182)
Supplement: Supplementary file 1 [file Table_1.DOCX]

***Supplementary Table 1.*** Frequency distribution of the baseline characteristic of the asthmatic patients (cases) and the healthy controls participated in the study.

| *Age group* | *Asthmatic patients*  *No (%)* | | *Healthy controls*  *No (%)* | | *Total*  *No (%)* |
| --- | --- | --- | --- | --- | --- |
|  | *male* | *female* | *male* | *female* |  |
| *5-15* | 14 (56.0) | 11 (44.0) | 14 (45.2) | 17 (54.8) | 56 (22.6%) |
| *16-25* | 7 (46.7) | 8 (53.3) | 7 (41.2) | 10 (58.8) | 32 (12.9%) |
| *26-35* | 10 (38.5) | 16 (61.5) | 12 (44.4) | 15 (55.6) | 53 (21.4%) |
| *36-45* | 7 (38.9) | 11 (61.1) | 5 (55.6) | 4 (44.4) | 27 (10.9%) |
| *46-55* | 5 (38.5) | 8 (61.5) | 4 (30.8) | 9 (69.2) | 26 (10.5%) |
| *56-65* | 4 (36.4) | 7 (63.6) | 5 (27.8) | 13 (72.2) | 29 (11.6%) |
| *>65* | 4 (33.3) | 8 (66.7) | 3 (23.1) | 10 (76.9) | 25 (10.1%) |
| *Total* | **51 (42.5)** | **69 (57.5)** | **50 (39.1)** | **78 (60.9)** | **248 (100%)** |
